# Ecology and Infection Dynamics of Multi-Host Amdoparvoviral and Protoparvoviral Carnivore Pathogens

**DOI:** 10.3390/pathogens9020124

**Published:** 2020-02-15

**Authors:** Marta Canuti, Melissa Todd, Paige Monteiro, Kalia Van Osch, Richard Weir, Helen Schwantje, Ann P. Britton, Andrew S. Lang

**Affiliations:** 1 Department of Biology, Memorial University of Newfoundland, 232 Elizabeth Ave., St. John’s, NL A1B 3X9, Canada; 2 British Columbia Ministry of Forests, Lands, Natural Resource Operations, and Rural Development, Coast Area Research Section, Suite 103-2100 Labieux Rd., Nanaimo, BC V9T 6E9, Canada; melissa.todd@gov.bc.ca (M.T.); paigemonteiro1@gmail.com (P.M.); kaliavanosch8@gmail.com (K.V.O.); 3 British Columbia Ministry of Environment and Climate Change Strategy, PO Box 9338 STN Prov Govt, Victoria, BC V8W 9M2, Canada; rich.weir@gov.bc.ca; 4 British Columbia Ministry of Forests, Lands, Natural Resource Operations and Rural Development, Wildlife Health Program, Wildlife and Habitat Branch, 2080 Labieux Rd., Nanaimo, BC V9T 6J9, Canada; helen.schwantje@gov.bc.ca; 5 Animal Health Center, British Columbia Ministry of Agriculture, 1767 Angus Campbell Rd., Abbotsford, BC V3G 2M3, Canada; ann.p.britton@gov.bc.ca

**Keywords:** Aleutian mink disease virus, canine parvovirus 2, feline panleukopenia virus, parvovirus, virus epidemiology, virus ecology, virus evolution, wildlife, mustelids

## Abstract

*Amdoparvovirus* and *Protoparvovirus* are monophyletic viral genera that infect carnivores. We performed surveillance for and sequence analyses of parvoviruses in mustelids in insular British Columbia to investigate parvoviral maintenance and cross-species transmission among wildlife. Overall, 19.1% (49/256) of the tested animals were parvovirus-positive. Aleutian mink disease virus (AMDV) was more prevalent in mink (41.6%, 32/77) than martens (3.1%, 4/130), feline panleukopenia virus (FPV) was more prevalent in otters (27.3%, 6/22) than mink (5.2%, 4/77) or martens (2.3%, 3/130), and canine parvovirus 2 (CPV-2) was found in one mink, one otter, and zero ermines (N = 27). Viruses were endemic and bottleneck events, founder effects, and genetic drift generated regional lineages. We identified two local closely related AMDV lineages, one CPV-2 lineage, and five FPV lineages. Highly similar viruses were identified in different hosts, demonstrating cross-species transmission. The likelihood for cross-species transmission differed among viruses and some species likely represented dead-end spillover hosts. We suggest that there are principal maintenance hosts (otters for FPV, raccoons for CPV-2/FPV, mink for AMDV) that enable viral persistence and serve as sources for other susceptible species. In this multi-host system, viral and host factors affect viral persistence and distribution, shaping parvoviral ecology and evolution, with implications for insular carnivore conservation.

## 1. Introduction

Mustelids (family: Mustelidae) are a diverse group of small mammalian carnivores with characteristic elongate bodies. There are 58 known species of mustelids and these include otters, mink, martens, fisher, ferrets, ermine (aka short-tailed weasel), badgers, and wolverine. They are widely distributed geographically across America, Africa and Eurasia, and they occupy a diverse range of habitats, from deserts to oceans. Their closest relatives are the mammals of the New World family Procyonidae (raccoons), from which they diverged approximately 28.7 million years ago, and they belong to the same superfamily (Musteloidea) that also includes skunks and red pandas [1,2].

**Figure 1:**
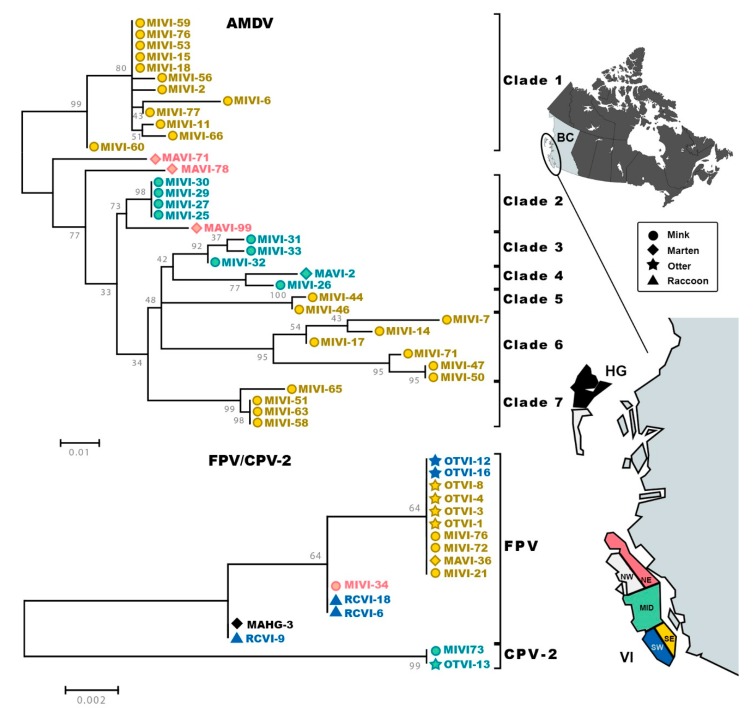
Molecular epidemiology of amdoparvoviruses (top) and protoparvoviruses (bottom) in insular BC. Phylogenetic trees are based on partial NS1 (for AMDV) and VP2 (for FPV and CPV-2) sequences and were obtained with the maximum-likelihood method [48], based on the HKY (for AMDV +G, +I) model [51], identified as the best-fitting model after the model test analysis, using MEGA 7 [46]. Branch support (1000 bootstrap iterations) is provided next to nodes. Strains are labelled with a shape indicating the host in which the virus was found (circle for mink, diamond for marten, star for otter and triangle for raccoon) and color-coded corresponding to the sampling area as illustrated on the map on the right. The position of British Columbia (BC) within Canada is shown on the top while the positions of Vancouver Island (VI) and the Haida Gwaii archipelago (HG) off the coast of BC are shown at the bottom. The subregions of VI indicated on the map (SE, south-east; SW south-west; MID, middle-island; NE, north-east; NW, north-west) correspond to those in Table 2. Maps were created with Mapchart.net ©.

British Columbia (BC) is the most westerly province of Canada and it includes numerous islands in the Pacific Ocean, including the large Vancouver Island (VI), only 2.5 km from the mainland at its closest point, and the more northerly Haida Gwaii archipelago (HG), located approximately 80 km from the mainland (Figure 1). Coastal glacial refugia, geographic isolation, and fragmentation have produced insular endemic populations of mustelids that are the best represented taxa among the carnivore communities of these coastal island systems [3]. Pacific marten (*Martes caurina*), typical of the North American coast and distinct from the mainland American pine marten (*Martes americana*) [4], are widely distributed, while ermines (*Mustela erminea*) are considered at risk [5,6,7]. While ermines, martens and North American river otters (*Lontra canadensis*) are found on both VI and HG [8], American mink (*Neovison vison*) are native only to VI [9] and, given the previous presence of fur farms, the probability of past hybridization of farmed mink and wild stocks is high. Finally, raccoons (*Procyon lotor*) are native to VI and were introduced to HG in the 1940s [10,11] while striped skunks (*Mephitis mephitis*), abundant on the mainland portion of BC, are absent from both island systems. Hereafter, we use marten, otter and mink to mean Pacific marten, North American river otter, and American mink, respectively.

Several different pathogens and parasites can affect mustelids and the most well studied are those that can frequently be found in mink on fur farms. These include bacteria such as *Pseudomonas aeruginosa*, which causes hemorrhagic pneumonia, and *Clostridium botulinum*, the causative agent of botulism, and viruses such as canine distemper virus and several different parvoviruses [12,13,14]. Parvoviruses (family *Parvoviridae*) are small, single-stranded DNA viruses with genomes of approximately 5 kb that include two main open reading frames (ORFs), one encoding the viral non-structural proteins (NS) and one encoding the viral capsid proteins (VP) [15]. Viruses from three different parvoviral genera, *Amdoparvovirus*, *Bocavirus*, and *Protoparvovirus*, have been found in mustelids [16,17,18,19,20].

Aleutian mink disease virus (AMDV, species *Carnivore amdoparvovirus 1*, genus *Amdoparvovirus*) is probably the most well-known virus of mink as it causes Aleutian disease, a chronic wasting syndrome characterized by high mortality and reproduction reduction in farmed mink [18]. Currently circulating AMDV strains probably originated in North America, but viruses have been diffused worldwide as a consequence of international animal trading and similar viral strains now circulate on farms and surrounding areas around the world [18,21,22]. Furthermore, other viruses within the genus *Amdoparvovirus* have recently been discovered that can infect related carnivores, such as skunk amdoparvovirus (SKAV), red panda amdoparvovirus (RpAPV), raccoon dog and fox amdoparvovirus (RFAV), gray fox amdovirus (GFAV) and red fox fecal amdovirus (RFFAV) [23,24,25,26,27]. Because these viruses replicate in macrophages and viral entry is likely mediated by cellular Fc receptors recognizing antibody covered viral particles, cross-species transmission is common [18]. For example, AMDV has been detected in several different mustelids, cross-species transmission between mink and skunk has been documented for both AMDV and SKAV, and RFAV can infect animals within different genera of the family Canidae [18,25,26].

Mink enteritis virus (MEV) causes a disease characterized by hemorrhagic enteritis associated with leukopenia, which is particularly severe in younger individuals [16]. MEV belongs to the species *Carnivore protoparvovirus 1* (genus *Protoparvovirus*) together with two other viruses that cause similar symptoms in canines and felines, canine parvovirus 2 (CPV-2) and feline panleukopenia virus (FPV); CPV-2 and FPV are the representatives of the two homonymous main lineages within this viral species. While FPV-like viruses, including MEV, have been circulating in wild and domestic animals for decades, CPV-2 emerged as a dog pathogen during the late 1970s as a variant of FPV or of a closely related virus [16]. These viruses have been identified in a wide array of hosts, such as domestic cats and dogs, wildcats, lynx, cougars, raccoons, skunks, wolves, foxes, coyotes, and many others [16,28,29,30,31,32,33,34,35]. Host specificity is determined in part by the affinity of viral capsid proteins to the cellular receptor (transferrin receptor type 1) and single amino acid mutations in the capsid protein can determine whether a host is susceptible to the virus [36,37]. Interestingly, FPV cannot infect canines and the original CPV-2 variant, now thought to be extinct, could not infect felines, while the currently circulating forms of CPV-2 (known as CPV-2a, b, and c) can infect both canines and felines [16,38]. Wildlife, and in particular raccoons, are believed to have played a crucial role in the emergence and evolution of CPV-2 and its variants [16,36].

Finally, in recent years, a novel protoparvovirus was identified in sea otter (*Enhydra lutris*) (sea otter parvovirus, SOP) and two novel bocaparvoviruses (genus *Bocaparvovirus*) were found in mink (mink bocavirus) and American pine marten (pine marten bocavirus), but our understanding about these viruses is still very limited and we have no knowledge about the distribution and impact of these viruses in wildlife and whether they can infect multiple hosts [17,19,20].

When dealing with such multi-host and multi-pathogen systems, molecular epidemiology and phylogenetic analyses can help clarify virus–host interaction dynamics. This is especially important for viruses of carnivores, particularly those of potential significance for vulnerable insular populations and for domestic and captive animal health, as most of these pathogens are not restricted to one single host, and over 90% of domestic carnivore pathogens infect multiple hosts [39]. For multi-host pathogens, combining these methods with the study of host ecology is fundamental in defining cross-species transmission dynamics, determining how hosts enable pathogen persistence and identifying which hosts, among epidemiologically connected and susceptible populations, are crucial for this persistence [40,41].

In this study, we performed molecular surveillance of parvoviruses circulating in mustelids of insular BC, focusing on the two most well-known viral species *Carnivore protoparvovirus 1* and *Carnivore amdoparvovirus 1*. We sequenced the identified viral strains and studied the molecular epidemiology and evolution of these viruses in their hosts in an attempt to acquire a better understanding of viral ecology and mechanisms behind parvoviral maintenance and cross-species transmission in wildlife.

## 2. Materials and Methods

### 2.1. Sample Collection

Samples for this study originated from wild animals that were either dead prior to submission, killed by licensed BC trappers for commercial purposes, killed on roads or farms, or in the case of ermines, incidentally caught in licensed traps and submitted for Compulsory Inspection on VI and HG (Province of BC Trapping Regulations). At necropsy, morphometric parameters (body length, chest girth, and weight) as well as sex were recorded for each animal. Age class (juvenile or adult) was accurately determined for marten by assessing temporal muscle coalescence [42] and estimated for other species based on relative size, weight and tooth wear. A gross examination of the internal organs was then performed, and the overall nutritional condition was assessed based on internal body fat depositions using a relative 4 class descriptive scale (poor, fair, good, excellent) adapted from [43]. Death year and date, if available, and collection location were also recorded. Spleens were then removed, stored individually in 4 ounce plastic Whirl-Pak bags, frozen at −20 °C, and shipped to Memorial University for molecular screening. This study was carried out in accordance with guidelines of the Canadian Council on Animal Care, with an approved protocol (15-04-AL) by the Memorial University Institutional Animal Care Committee, and an approved BC Animal Care protocol.

In total, spleens were collected from 256 mustelids that died between 2002 and 2019, including 130 Pacific martens, 77 American mink, 27 ermines, and 22 river otters. All animals originated from islands off the coast of BC: 226 were from VI, 26 were from HG (12 from Graham Island and 14 from Moresby Island), 3 were from Minstrel Island, and 1 was from Quadra Island. Details about sampled animals are summarized in Table S1, Supplementary Results.

### 2.2. Screening and Sequencing

DNA was isolated from approximately 10 mg of tissue using the DNeasy Blood and Tissue Kit (Qiagen) and used for molecular screening. All samples were screened for protoparvoviruses (CPV-2 and FPV) and amdoparvoviruses (all known species) as described previously [21,30,31], but with the addition of a second amdoparvovirus PCR with primers SKAV-1F (CCAACAAGTAATGACACCWTGG) and AMDO-6R (equimolar mixture of CTCCAGYAAAGTAACTACC and GTCCACCAACAAAGTAACTACC). This second primer pair, designed within a highly conserved genomic region identified based on sequence alignments of all currently known amdoparvoviruses, can potentially be used to amplify yet unknown viral lineages or species. Otters were screened for the SOP using previously published primers [20] and 104 samples from mink (N = 52) and martens (N = 52) were screened by heminested PCR for the novel carnivore bocaparvoviruses with primers MuBoV_F1 (RCAGACACRCTATACAACAATG), MuBoV_R1 (TCYTCYCCTGTTCGYAGCAC), and MuBoV_F2 (TSTGTGATGGSTCGCATC). Positives were confirmed by sequencing the obtained amplicons and the complete genomes were obtained from a selection of strains by performing overlapping PCRs and sequencing. All primers used for complete genome sequencing are available upon request.

### 2.3. Sequence Analysis

Sequences obtained in this study were compared to all full NS1 and VP2 coding sequences of *Carnivore protoparvovirus 1* (CPV-2 and FPV), and *Carnivore amdoparvovirus 1* and *4* (AMDV and SKAV, respectively) available in the GenBank database (https://www.ncbi.nlm.nih.gov) as of 14 October, 2019 (N = 397 and N = 2082 for protoparvovirus NS1 and VP2, respectively; N = 301 and N = 288 for amdoparvovirus NS1 and VP2, respectively). Sequences were aligned using Clustal W [44] and alignments were inspected for recombination using RDP5 [45] as previously described [21], and used for phylogenetic inference using MEGA 7 [46]. Trees were built with the neighbor-joining (large datasets) and maximum-likelihood (small datasets) methods [47,48] using the best model for distance estimation identified by a modeltest analysis and robustness of clades was assessed with a bootstrap analysis (1000 replicates) [49]. Genetic similarities (1 - p-distance) between strains and between and within clades were calculated with MEGA 7.

### 2.4. Statistical Analyses

Differences between viral positivity rates in different host populations (number of positive animals over the total number of individuals) were evaluated for statistical significance using the Mid-p exact test, while an analysis of variance (ANOVA) was used to compare continuous variables overall and after adjusting for sex and age. Both analyses were performed with OpenEpi [50] and *p*-values ≤0.05 (2-tailed tests) were considered significant.

### 2.5. Data Availability

All sequences obtained in this study have been deposited in the GenBank database under accession numbers MN862728-MN862749 for complete genomes and MN862700-MN862727 for partial sequences.

## 3. Results

### 3.1. Viral Prevalence and Host Distributions

In total, 19.1% of the screened animals (49/256) were positive for at least one virus and one co-infection was identified. Amdoparvoviruses were found in mink and martens, while protoparvoviruses were identified in mink, martens, and otters. No viruses were detected in ermines and no sample tested positive for bocaparvoviruses. Mink had the highest total viral prevalence rate (46.8%), followed by otters (31.8%), and martens (4.6%). Both viral genera were found on VI, while only protoparvoviruses were found in HG (Graham Island).

All 36 detected amdoparvoviruses were identified as AMDV and prevalence was significantly higher (*p* < 0.001) in mink (41.6%), compared to martens (3.1%). Furthermore, AMDV was significantly more prevalent than protoparvoviruses in mink (41.6% vs. 6.5%, *p* < 0.001), but not in martens (3.1% vs. 1.5%, *p* = 0.45). Conversely, protoparvoviruses were significantly more prevalent in otters than in both mink (31.8% vs. 6.5%, *p* < 0.005) and martens (31.8% vs. 1.5%, *p* < 0.001).

Among the 14 identified protoparvoviruses, 85.7% (12/14) were classified as FPV, which were found in mink, martens, and otters in VI and one marten in HG. The remaining two protoparvoviruses were classified as CPV-2 and these were found in one mink and one otter from VI. One AMDV-FPV co-infection was identified in a mink. Screening results are summarized in Table 1. Since martens originated from different island regions, both VI and HG, we decided to keep the two populations separate as there are likely no contacts between them.

AMDV prevalence was higher, although not significantly (*p* = 0.1), in adult mink (45.5%) compared to juveniles (18.2%), while two of the four positive martens were adults. On the contrary, protoparvoviral prevalence was significantly higher in juvenile (62.5%) compared to adult (14.3%) otters (*p* < 0.05) and the two protoparvovirus-positive martens were juveniles. Interestingly, all protoparvovirus-positive mink (N = 5) were adult males. For all viruses, no significant differences were identified between males and females or across different years.

No associations between body conditions and viral infection were identified for AMDV (including enlarged spleens) or for protoparvoviruses in martens and otters. Although protoparvovirus-positive mink were significantly heavier (*p* = 0.01) than non-infected animals, this difference was lost when corrected for age and sex (*p* = 0.07). Among adult male mink, non-infected animals were significantly longer than protoparvovirus-positive animals (*p* = 0.01), but the standard deviation of the infected group was high and indicative of poor homogeneity in this group. These results are shown in more detail in Tables S2–S5, Supplementary Results.

### 3.2. Spatial Distributions

To test whether the difference in viral prevalence among the various hosts was influenced by sample collection location, we divided VI into five ecologically separated subregions. As shown in Table 2 and Figure 1, AMDV was present throughout all subregions of VI sampled, except for the west side of the island, where only a few samples were available due to limited accessibility. In mink, prevalence varied considerably across locations, with rates reaching almost 90% in the middle-island region of VI, where the positivity rate was significantly higher than anywhere else (*p* < 0.02) (Table 2, Table S2, Supplementary Results). Similar to what was observed in the total population, in both locations where AMDV was highly prevalent in mink, AMDV was significantly more prevalent in mink with respect to martens (south-east and middle-island regions of VI). This was also true for three specific locations within the south-east VI subregion, Copper Canyon (6/11, 54% vs. 0/14, *p* < 0.01), Mesachie Lake (7/18, 38.9% vs. 0/18, *p* < 0.01), and Ladysmith-Mckay Peak area (5/14, 35.7% vs. 0/15, *p* < 0.05) (Tables S2 and S3, Supplementary Results). Finally, no AMDV-positive otters were found in areas with high AMDV prevalence in mink.

Protoparvoviruses were not as geographically widespread and were most prevalent in the south-east part of VI, where human density is the highest (Table 2, Figure 1). As observed for the total population, within the south-east subregion, where samples from all three host species were available, protoparvoviral prevalence was significantly higher in otters compared to both mink and martens. Unfortunately, the numbers of samples from each species with definite locations within the subregions were not adequate for meaningful comparisons (Tables S2, S3 and S5, Supplementary Results). However, within the south-east subregion, protoparvovirus-positive mink were only found at locations where protoparvoviruses were also found in otters.

### 3.3. Molecular Epidemiology of Parvoviruses in Insular BC

To study whether there was a correlation between virus type and host or location, all amplicons obtained during screening were sequenced and phylogenetic trees were built with all parvoviral sequences identified so far on VI and HG, which include sequences from this and a previous study that identified protoparvoviruses in VI raccoons [30].

On average, AMDV sequences were 94.9% identical to each other (range: 90.8%–100%) and no evidence of recombination was found. Phylogenetic analysis (Figure 1) revealed the presence of seven main supported clades (bootstrap >70), whose existence was also supported by a within-between clades distance analysis, where the average pairwise distance between sequences within one clade was lower than the average pairwise distances between sequence pairs from different clades (Table S6, Supplementary Results). These clades included all but two sequences from martens, MAVI-78 and MAVI-71, which seemed to occupy an intermediate position between clade 1 and all other sequences. The other two sequences from viruses identified in martens fell within clades that also contained sequences from mink (clades 2 and 4). There was an evident geographic segregation of strains, illustrated by the clustering of sequences according to sampling locations. The subregion south-east VI presented the highest viral diversity with the presence of viruses from 4 different clades, three of which (clades 5, 6, and 7) were more closely related with respect to a more distant one (clade 1). Only one clade (clade 2) included sequences from two different, although adjacent, subregions.

FPV sequences were, on average, 99.7% identical to each other (range: 99.2%–100%), while the two CPV-2 sequences were identical. No evidence of recombination was found. In the phylogenetic tree (Figure 1) the two main clades corresponding to CPV-2 and FPV could be observed, and the FPV sequences were further divided in three different variants. No pattern could be observed with respect to host distribution as each clade included viruses identified in different hosts. Most strains were identified in animals sampled in the two adjacent southern subregions of VI (south-east and south-west). The only two exceptions were a strain found in a marten from HG and one found in a mink from the north-east subregion of VI, both clustering with sequences from raccoons from the southern part of VI. Therefore, there was no evident geographic segregation of strains.

### 3.4. Molecular Epidemiology of Parvoviruses in a Global Perspective

The full coding sequences of a selection of viruses were obtained to study the relationship between viruses found in BC and those identified in other parts of the world. Since viral proteins are generated by translating different messenger RNAs produced by alternative splicing, viral ORFs were determined by *in silico* splicing following what was experimentally demonstrated for both parvoviral genera. In detail, amdoparvoviruses and protoparvoviruses possess three and two NS proteins, respectively, and they both possess two VP proteins. In both cases all NS proteins share the same N-terminal sequence, 60 amino acids in length in the case of amdoparvoviruses and 87 in the case of protoparvoviruses, but have unique C-terminal sequences, while the VP proteins share the same C-terminal sequences but they begin at a different start codon. While in amdoparvoviruses all ORF, except VP2, are generated after splicing and all messenger RNAs are transcribed from a single promoter, in protoparvoviruses only the VP1 and NS2 ORFs are generated after splicing and structural and non-structural protein expression is directed by two different promoters [15,18,38,52,53,54].

**Figure 2:**
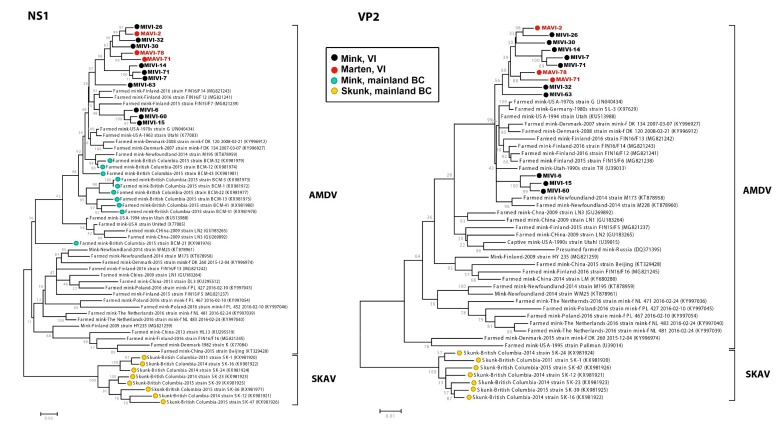
Phylogenetic analyses of AMDV and SKAV complete NS1 and VP2 genes. Phylogenetic trees were obtained with the maximum-likelihood method [48], based on the GTR (+G for NS1, +G and +I for VP2) model [55], identified as the best-fitting model after the model test analysis, using MEGA 7 [46]. The outcome of the bootstrap analysis [49] is shown next to the nodes. Strains identified in BC are labelled with circles colored corresponding to the host in which they were identified (black for VI mink, red for VI martens, teal for farmed mink, and yellow for mainland skunks).

**Figure 3:**
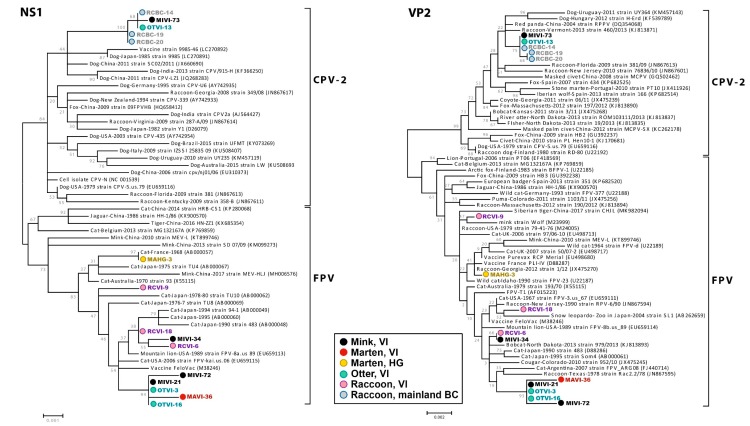
Phylogenetic analyses of CPV-2 and FPV complete NS1 and VP2 genes. Phylogenetic trees were obtained with the maximum-likelihood method [48], based on the HKY +G [51] and T92 +G [56] models for NS1 and VP2, respectively, identified as the best-fitting models after the model test analysis, using MEGA 7 [46]. The outcome of the bootstrap analysis [49] is shown next to the nodes. Strains identified in BC are labelled with circles colored corresponding to the host in which they were identified (black for VI mink, red for VI marten, yellow for HG marten, teal for VI otters, grey for mainland raccoons, and purple for VI raccoons).

After determining ORF sequences, phylogenetic analyses were performed. Initially, neighbor-joining trees were built for both the complete NS1 and VP2 genes of both amdoparvoviruses and protoparvoviruses including all sequences available in GenBank (Figures S1–S4, Supplementary Results). Subsequently, a subset of these sequences was used to construct maximum-likelihood phylogenies (Figure 2 and Figure 3).

The almost complete genome was obtained for 13 AMDV strains (10 from mink and 3 from martens). Unfortunately, because of the high G/C content and low viral load, for some of the viruses, we could not fully resolve the area at the beginning of the VP2 protein-coding region corresponding to the glycine stretch. This area, which is highly variable in size and causes issues during sequence alignments, was removed from all following analyses. Some differences were observed across the sequenced genomes. Due to the variable position of the stop codon, the NS3 proteins were variable in length (68–92 amino acids), but NS3 proteins of similar length were previously annotated in GenBank. Four sequences (MAVI-71, MAVI-78, MIVI-6, and MIVI-63) presented one amino acid deletion in the VP1/VP2 proteins. Finally, the three viruses from clade 1 in Figure 1 for which we obtained the complete genomes contained a 5 nt insertion between the two ORFs.

The complete genome sequences were 93.8%–98.8% identical and we found no evidence of recombination among the BC strains. Phylogenetic analyses performed with the NS1 gene (Figure 2 and Figure S1, Supplementary Results) showed that the identified viruses formed two different but closely related and highly supported clades (bootstrap ≥92) within the AMDV viral species. One of these clades corresponded to clade 1 in Figure 1 and included only sequences from mink sampled in proximal areas (south-east subregion of VI), while all other island BC sequences clustered together. Although some of the clades were less well supported, likely because the VP2 region is less powerful in resolving amdoparvoviral phylogenetic relationships [21], similar results were obtained with the VP2 gene (Figure 2 and Figure S2, Supplementary Results). Interestingly, in all trees two of the viral sequences identified from martens from the same area (MAVI-71 and MAVI-78) formed a highly supported clade. Finally, all island BC strains were closely related to viruses identified in mink farms worldwide, but closer to viruses identified in European farms than to viruses identified in mainland BC, including the AMDV strains from farmed mink and the SKAV strains from wild skunks.

Almost complete viral genomes were obtained for nine protoparvoviruses: the two CPV-2 strains (one from a VI mink and one from a VI otter) and seven FPVs (two from VI otters, three from VI mink, one from a VI marten, and one from a HG marten). All FPVs possessed the molecular markers in VP2 that characterize FPV strains, while the two CPV-2 strains could be classified as CPV-2a, but they possessed the same mutation at position 305 (VP2-305His) previously observed in viruses from BC raccoons [30] (Table S6, Supplementary Results). This mutation has previously been observed in raccoon viruses that were defined as intermediate between CPV-2 and CPV-2a and that, it has been hypothesized, facilitated the emergence of CPV-2a in dogs [28,30]. Also similar to other BC strains from raccoons, the two CPV-2 strains possessed an Asp in position 300 of the VP2 protein, a key residue for determining host range, and a VP2-301Ala (found only in BC sequences and in a sequence from 2008 from a palm civet in China). Interestingly, while various amino acids have been observed at position 300 in FPV strains from mustelids [37], all FPVs from this study possessed a combination of 300Ala and 301Thr, typical of feline strains and of BC raccoon strains.

The complete FPV genomes were 99.2%–100% identical to each other, while the two CPV-2 strains were almost identical (99.9%). No evidence of recombination was found. In all phylogenetic trees (Figure 3 and Figures S3–S4, Supplementary Results), the two CPV-2 strains identified in this study from a mink and an otter formed a highly supported cluster together with the other three CPV-2 strains previously identified in raccoons from mainland BC, suggesting the circulation of a local CPV-2 strain. On the contrary, FPV sequences were distributed within different clades in the trees, indicating the co-circulation of different strains. One of these clades, which was highly supported (bootstrap ≥93), seemed to be unique to VI as it included exclusively five strains that were all from this island but identified in three different mustelids (mink, marten and otter). Furthermore, one sequence from a mink and one from a raccoon consistently clustered together, while the remaining sequences, two from raccoons from southern VI and one from a marten from HG, were scattered in other branches.

## 4. Discussion

The viral family *Parvoviridae* includes viruses infecting a very wide range of hosts, including both vertebrates and invertebrates that inhabit diverse ecosystems, spanning from terrestrial to aquatic and marine environments [15]. Within the subfamily *Parvovirinae*, which so far includes only viruses of vertebrates, viruses within three genera are known to infect terrestrial carnivores, including mustelids. Nevertheless, several studies highlight how our knowledge of (parvo)virus diversity is still very limited as many novel parvoviruses, including those infecting carnivores, are being discovered [17,19,20,23,25,26,27]. Furthermore, for many of the already known viruses we have a very limited understanding about host distribution, especially among wildlife, as they are predominantly studied in domestic or captive populations.

In this study, in an attempt to clarify aspects of viral maintenance, ecology, and virus–host and cross-species transmission dynamics among wildlife, we investigated all currently known mustelid parvoviruses and acquired epidemiological and genetic data about viruses circulating among the wild mustelids of insular BC. With the exception of species translocations and introductions, VI is a semi-closed environment with populations of mink, martens, otters, and raccoons that can disperse between the island, smaller adjacent islands, and the neighboring mainland, and ermines that are unlikely to disperse between the island and the mainland. Exclusive of species introductions, HG is a closed system for mammals given its much greater distance from the mainland. Acquiring knowledge about infectious disease exposure, viral persistence and transmission, and consequent potential for disease, are of great significance for conservation concerns for wild insular endemic carnivore populations not only in BC, but also in similar systems in other parts of the world, especially since there are well documented cases of island population declines resulting from infectious diseases [57]. Furthermore, monitoring disease transmission from introduced species to insular endemic wildlife populations is a major conservation concern in BC and around the world, especially since many of these populations are at risk due to the impacts of human settlement, land use, and climate change [57,58,59].

### 4.1. Ecology and Evolution of Parvoviruses in Insular BC

Among the eight viral species targeted by our screening methods, three different viruses, AMDV, CPV-2, and FPV, were identified, with several co-circulating viral lineages. Two different but closely related lineages of AMDV were found in mink and martens, one CPV-2 lineage was identified in mink and otters, and three different lineages of FPV were found in mink, martens, and otters. Furthermore, two additional lineages of FPV were previously reported in raccoons in the same area [30].

It is not surprising that some of the viruses under investigation were not observed as they may be restricted to distant areas [17,24], to more distantly related hosts [23,24], or to captive environments [19,25,27]. However, we could also not detect the presence of SKAV, which is the closest known relative to AMDV, known to circulate with high prevalence rates among skunks of mainland BC, and for which spillover to mink has been documented [26]. This indicates that either SKAV never reached these BC islands or that skunks, absent from both VI and HG, are the only maintenance hosts enabling viral persistence and mink is just a dead-end spillover host. This could be clarified with epidemiological investigations of wild mink that live in areas where SKAV is highly prevalent. Finally, no novel amdoparvoviral species or lineages were identified, although the PCRs we used were designed to detect potentially novel viruses. We cannot, however, exclude the presence of yet unknown parvoviruses among these mustelid populations as viruses that are highly divergent from the currently known strains could have been missed.

Two different viruses, CPV-2 and FPV, were found in river otters. For both viruses, only one strain was identified, and it was included within a highly supported BC-specific clade, somewhat separated from the other protoparvoviruses. CPV-2 was certainly introduced locally in the otter population as the presence of these animals in coastal BC predates viral emergence in North America, which occurred in the late 1970s [38]. BC raccoons are likely the donor host as CPV-2 circulates at very high prevalence among these animals [30], while only one otter was found positive for this virus, and viruses found in raccoons and otters are monophyletic and genetically very close. Furthermore, all these CPV-2 strains possessed the same intermediate mutations previously observed in raccoons throughout North America (VP2-305His, VP2-300Asp) [30,36].

On the contrary, with a prevalence of almost 30%, FPV seems to be endemic in the BC river otter population, similar to what is observed in Eurasian otter (*Lutra lutra*) [34]. Interestingly, although they shared the same key host-determinant residues, 300Ala and 301Thr, the FPV strains we found in otters differed from those found in raccoons sampled in proximal regions. Nevertheless, it is likely that FPV can also be transmitted between otters and raccoons although further screening is required to verify this. We can, however, confirm cross-species transmission among the three investigated mustelid species for FPV, as viruses similar to those found in otters were also found in mink and martens. These viruses formed a highly supported clade that was fairly distant from all of the other FPVs, presumably reflecting a bottleneck with a longer evolutionary time to differentiate locally, especially considering that FPV is more genetically stable than CPV-2 [60]. However, the extent of our knowledge about the genetic diversity of these viruses in wildlife is far from complete and it is possible that related strains circulating elsewhere have simply not been identified yet.

A relatively high level of AMDV diversity was identified and the co-circulation of several different viral clades was observed. All the identified strains belong to two main clades and are highly similar to viruses previously identified in mink farms all around the world. Although there have not been any active mink farms on VI since 1999, there is still active farming in the lower mainland portion of BC. Mink farming on VI has been documented since 1922 [61] with up to 51 farms simultaneously active by 1940, geographically distributed almost exclusively along the south and eastern coasts of the island, with another five on the neighboring Gulf islands [62]. Animals were presumably imported from or exchanged with other farms during the years of operation. This likely led to the introduction of AMDV on the island and the consequent viral leak to wildlife. Mink exchange between farms has led to a dispersal of similar AMDV strains worldwide, which then differentiated locally into regional lineages [18]. Therefore, evolutionary dynamics of founder effects and genetic drift likely explain our observation of local farm-derived AMDV lineages among the wildlife of VI. Since the average longevity of mink in the wild is three years [9], this finding demonstrates that AMDV can efficiently perpetuate in the wild independent of farms. In fact, AMDV is now endemic in mink, with a prevalence of approximately 42%, while only 6.5% of these animals were infected with protoparvoviruses. The three protoparvoviral strains identified in mink, one CPV-2 and two FPV strains, were highly similar to strains from otters and racoons, providing evidence for potential viral transmission from these species, where infection prevalence is high, to mink.

Both protoparvoviruses and amdoparvoviruses were identified in Pacific martens, both at a low rate (approximately 3% and 2% for AMDV and FPV, respectively). Although the AMDV sequences found in martens were highly similar to those obtained from mink, indicating a common origin, two of these viruses formed their own clade. Unfortunately, we cannot conclude if these viruses were marten-specific strains as they were identified in the north-east subregion area from where no mink samples were available, and the divergence observed between these strains and the others could be due to geographic diversification. In fact, our data support a local diversification of strains with the consequent circulation of subregional specific strains. This is also confirmed by recent microsatellite analysis of the martens sampled for this study, which demonstrates substantial genetic differentiation between the north-east and south-east subregions, suggesting limited connectivity between populations in these subregions [63]. Furthermore, the same data show that two of the martens in our samples, including one of the two individuals infected with the marten-specific virus, were not related to other VI marten, suggesting possible marten dispersal to VI from adjacent islands or the mainland. It is therefore possible that this AMDV lineage was imported from other parts of BC and additional viral genetic data from areas neighboring VI would clarify this aspect.

While the FPV found in one VI marten was very similar to the FPVs circulating in otters and mink and therefore suggesting marten to be a spillover host, the FPV strain found in a marten from HG was unique to this host and was more similar to other FPV strains found elsewhere. Martens are native on HG and there is no natural exchange of animals between HG and the mainland because the archipelago, which was isolated after the last glaciation, is too far offshore [5]. Although this virus could be a direct descendant of older FPVs that have been circulating in HG for decades, this virus showed no evidence of genetic drift, which is peculiar for a virus that supposedly evolved in seclusion for a long time. Another introduction mechanism, such as spillover from domestic animals or from fur farms, should therefore be considered for this virus [41]. Indeed, three mink farms were reported active on Graham Island in HG between 1922 and 1940 that were situated in active settlements [64] and the FPV-positive marten was found just north of one of these settlements, Port Clements, killed crossing the road from the local landfill. Finally, another possible source of infection is raccoons, which were introduced to Graham Island in the 1940s and could have carried the virus [11]. It is likely that FPV circulates within the local populations of raccoons and river otters but, unfortunately, samples from these animals from HG were unavailable for this study and future efforts should be directed at identifying more HG strains from a larger number of hosts.

Finally, no parvoviruses were detected in ermines. This could be due to a lower chance for ermines to acquire parvoviral infections and perpetuate the virus, also given their presumed low densities on the islands, or because there are less opportunities for cross-species transmission. However, the number of ermine samples collected and tested was low, and we cannot draw definitive conclusions about lack of detection without further sampling.

### 4.2. Epidemiology and Ecology of Parvoviruses and Their Hosts

The viruses on which this study focuses belong to two different, but connected, parvoviral genera. *Amdoparvovirus* and *Protoparvovirus* are monophyletic and both include viruses capable of infecting a wide range of carnivore hosts that, in some cases, overlap. However, they are separated by important genetic differences and the mechanisms behind “host-non-specificity” differ considerably between the two [18,37,65]. We may, therefore, expect significant differences in viral ecology and cross-species transmission dynamics for these two groups.

Overall, approximately 20% of the tested animals were positive for at least one virus, with *Carnivore amdoparvovirus 1* being more prevalent than *Carnivore protoparvovirus 1* (14% vs. 5%). However, viral prevalence differed significantly between the different hosts: AMDV prevalence was the highest in mink (>40%), while protoparvoviral prevalence was the highest in otters (32%). Furthermore, while both viral species were found in martens and mink, AMDV was significantly more prevalent than protoparvoviruses in mink, but there was no difference in positivity rates in martens. Finally, only protoparvoviruses were found in otters and no viruses were found in ermines. This shows how, in a multi-host system, viral distribution can significantly vary between different host species, even if they share the same habitats.

Given their low abundance and use of a wide variety of habitat types [6], insular ermines have limited spatial overlap with larger mustelids, although those sampled for this study were captured in marten trap sets. However, insular Pacific martens, mink, and river otters overlap in the freshwater riparian and coastal marine interfaces and their diets may overlap. This is especially true for mink and otters, which exclusively exploit these aquatic environments, while in moist coastal environments martens are strongly associated with forests and prey in the streamside environments [66]. Raccoons commonly forage in streams and riparian areas and insular populations are frequent foragers in the intertidal and shallow subtidal marine interface [67], where they overlap with martens and river otters in HG and VI, and coastal mink on VI. This ecological overlap explains how viral exchange among the studied populations is possible. However, other factors, such as host susceptibility and viral transmission dynamics, may also influence viral spread among different wild populations.

Protoparvoviral prevalence was significantly higher in otters than in both mink and martens. Furthermore, viral genetic data showed that protoparvoviral exchange happens among four different hosts (mink, martens, otters, and raccoons), but this was not true for every host species pair. For example, no virus was identified that was shared between martens and raccoons. However, four of the six lineages we identified were represented by one or two strains, and it is possible that viral diversity is higher than we detected, and more extensive screening may reveal more cross-species transmission links. In comparison, all amdoparvoviruses identified in mink and martens were genetically very similar, but these viruses were not found in otters, even in areas with high AMDV prevalence. Furthermore, amdoparvoviral prevalence was consistently higher in mink compared to martens, in total and in locations where both were sampled at a relatively similar intensity. Overall, these data suggest that, although viral exchange does happen, it may not be as frequent as we could postulate based on habitat overlap and it may not be multidirectional. In other words, although cross-species transmission to spillover hosts is common, different maintenance hosts seem to be required for the persistence of different parvoviruses.

Interestingly, the demographics of infected individuals were different for both viruses in the different hosts. While AMDV was more frequently observed in adult mink, consistent with a model of chronic infection for amdoparvoviruses where antibodies enable viral replication [18], protoparvoviruses were significantly more prevalent in juvenile otters, consistent with a model of acute infection acquired early in life with the consequent development of protective immunity [16]. Surprisingly, all protoparvovirus-positive mink were adults. Although further studies involving a higher number of samples and a more accurate aging from teeth are required to confirm this aspect, it is possible that young mink are more susceptible to protoparvovirus infection relative to other mustelids and have a higher protoparvovirus-related mortality. Since a somewhat virus-tolerant population is required for viral maintenance, a postulated higher susceptibility of mink to these viruses might explain the higher prevalence of FPV in otters. Conversely, yet unknown host-associated factors may confer some protection to otters against AMDV and protect them from acquiring amdoparvoviral infections.

## 5. Conclusions

Overall, we observed the endemic circulation of three different parvoviruses from two different viral genera on large islands in coastal BC, with the co-circulation of several different lineages, both old and more recently introduced ones, and we found evidence of bottleneck events, founder effects, and genetic drift that generated regional lineages. Our prevalence and genetic data combined support a model where cross-species transmissions are frequent among mustelids and related animals. However, we could observe the presence of principal maintenance hosts for each virus (otters and raccoons for the protoparvoviruses and mink for AMDV) that we believe are required to ensure viral persistence and that are the main source of viruses for other susceptible species, where genetically related viruses circulate at a lower frequency. Furthermore, the likelihood for cross-species transmission between two host species is not the same for different viruses as there are epidemiological discrepancies between amdoparvoviruses and protoparvoviruses in the same two host species. For example, mink acquire FPV infection from otters, but otters do not acquire AMDV infection from mink. Finally, with regards to host distribution, we postulate that different viruses may not necessarily move bidirectionally, and dead-end spillover host species likely exist. In this multi-pathogen and multi-host system, the intricate interplay between viral characteristics, such as cell entry mechanism or environmental stability, and host factors, like susceptibility to infection and habitat overlap, affect viral persistence and distribution, ultimately shaping the viral ecology and evolution.

## Figures and Tables

**Table 1:** Parvoviral prevalence among different mustelid populations of insular British Columbia.

	Mink (VI)^1^ N = 77	Marten (VI) N = 107	Marten (HG) N = 23	Otter (VI) N = 22	Ermine N = 27	Total N = 256
**AMDV^2^**	32 (41.6%)	4 (3.7%)	0	0	0	36 (14.1%)
**CPV-2**	1 (1.3%)	0	0	1 (4.5%)	0	2 (0.8%)
**FPV**	4 (5.2%)	2 (1.9%)	1 (4.4%)	6 (27.3%)	0	12 (4.7%)
**SOP**	/^3^	/	/	0	/	0
**BoV**	0	0	/	/	/	0
**Total**	36 (46.7%)	6 (4.6%)	1 (4.4%)	7 (31.8%)	0	49 (19.1%)

^1^ VI: Vancouver Island; HG: Haida Gwaii. One mink was from Quadra Island, ermines were from VI, HG and Minstrel Island; ^2^ AMDV: Aleutian mink disease virus; CPV-2: canine parvovirus 2; FPV: feline panleukopenia virus; SOP: sea otter parvovirus; BoV: bocaparvoviruses. ^3^ A/indicates that no samples were screened for this virus as only a subset of samples was tested (22 otters for SOP; 52 mink and 52 martens for BoV).

**Table 2:** Infection rates in the different hosts for the different viruses in the various sampled locations.

	AMDV		FPV/CPV-2		
	Mink N (% pos)	Marten N (% pos)	Mink N (% pos)	Marten N (% pos)	Otter N (% pos)
**SE VI ^1^**	60 (40) *	47 (0) *	60 (6.7) **	47 (2.1) *	14 (35.7) **^,^*
**SW VI**	3 (0)	/	3 (0)	/	8 (25)
**MID VI**	9 (88.9) ***	5 (20) ***	9 (0)	5 (0)	/
**NE VI**	4 (0)	38 (7.9)	4 (25)	38 (0)	/
**NW VI**	/^3^	17 (0)	/	17 (0)	/
**HG^2^**	/	23 (0)	/	23 (4.3)	/

^1^ Vancouver Island (VI) subregions: SE, south-east; SW south-west; MID, middle-island; NE, north-east; NW, north-west. ^2^ HG: Haida Gwaii. ^3^ A/indicates that no samples were collected in that region. Locations where prevalence was significantly higher (*p < 0.02*) are indicated in bold. * *p* ≤ 0.001, ** *p* ≤ 0.01, *** *p* ≤ 0.05.

## Supplementary Materials

The following are available online at https://www.mdpi.com/2076-0817/9/2/124/s1. Table S1: Summary details for animals screened in this study; Table S2: Parvovirus screening results for mink; Table S3: Parvovirus screening results for Vancouver Island Pacific martens; Table S4: Protoparvovirus screening results for Haida Gwaii Pacific martens; Table S5: Protoparvovirus screening results for river otters; Table S6: Percentage identity (1 - p-distance) within and between AMDV taxa identified in insular British Columbia; Table S7: Amino acid mutations in VP2 of the prototype strains (FPV, CPV, CPV-2a, CPV-2b and CPV-2c) and of the viruses characterized in this study; Figure S1: Phylogenetic analysis of the complete amdoparvoviral NS1 gene; Figure S2: Phylogenetic analysis of the complete amdoparvoviral VP2 gene; Figure S3: Phylogenetic analysis of the complete protoparvoviral NS1 gene; Figure S4: Phylogenetic analysis of the complete protoparvoviral VP2 gene.
