# Supplementary material for: Ecology and Infection Dynamics of Multi-Host Amdoparvoviral and Protoparvoviral Carnivore Pathogens
Source: Pathogens. 2020 Feb 15;9(2):124. doi: 10.3390/pathogens9020124 (PMC7168296; doi:10.3390/pathogens9020124)
Supplement: Supplementary file 1 [file pathogens-09-00124-s001.pdf]

# Ecology and infection dynamics of multi-host amdoparvoviral and protoparvoviral carnivore pathogens

Marta Canuti, Melissa Todd, Paige Monteiro, Kalia Van Osch, Richard Weir, Helen Schwantje, Ann P. Britton, Andrew S. Lang

**Content**

**Table S1. Summary details for animals screened in this study** .......... 2

**Table S2. Parvovirus screening results for mink** .......... 3

**Table S3. Parvovirus screening results for Vancouver Island Pacific martens** .......... 4

**Table S4. Protoparvovirus screening results for Haida Gwaii Pacific martens** .......... 5

**Table S5. Protoparvovirus screening results for river otters** .......... 5

**Table S6. Percentage identity (1 - p-distance) within and between AMDV taxa identified in insular British Columbia** .......... 6

**Table S7. Amino acid mutations in VP2 of the prototype strains (FPV, CPV, CPV-2a, CPV-2b and CPV-2c) and of the viruses characterized in this study** .......... 6

**Figure S1. Phylogenetic analysis of the complete amdoparvoviral NS1 gene** .......... 7

**Figure S2. Phylogenetic analysis of the complete amdoparvoviral VP2 gene** .......... 8

**Figure S3. Phylogenetic analysis of the complete protoparvoviral NS1 gene** .......... 9

**Figure S4. Phylogenetic analysis of the complete protoparvoviral VP2 gene** .......... 10

**Table S1. Summary details for animals screened in this study.**

| | American mink | Pacific marten | Ermine | River otter | Total |
|---|---|---|---|---|---|
| **Total** | 77 | 130 | 27 | 22 | 256 |
| **Sex** | | | | | |
| Female | 21 | 60 | 6 | 10 | 97 |
| Male | 56 | 70 | 21 | 12 | 159 |
| **Age** | | | | | |
| Juvenile | 11 | 65 | 2 | 8 | 86 |
| Adult | 66 | 65 | 10 | 14 | 155 |
| Unknown | /[1] | / | 15 | / | 15 |
| **Location** | | | | | |
| Vancouver Island | 76 | 107 | 21 | 22 | 226 |
| Haida Gwaii, Graham Island | / | 9 | 3 | / | 12 |
| Haida Gwaii, Moresby Island | / | 14 | / | / | 14 |
| Minstrel Island | / | / | 3 | / | 3 |
| Quadra Island | 1 | / | / | / | 1 |
| **Death year** | | | | | |
| 2002-2015 | / | / | 18 | / | 18 |
| 2016 | / | 79 | 1 | / | 80 |
| 2017 | 21 | 37 | / | / | 58 |
| 2018 | 40 | 14 | 2 | / | 56 |
| 2019 | 16 | / | 6 | 22 | 44 |
| **Body condition** | | | | | |
| Excellent | 6 | 4 | / | 1 | 11 |
| Good | 32 | 40 | 6 | 3 | 81 |
| Fair | 29 | 73 | 14 | 15 | 131 |
| Poor | 10 | 13 | 7 | 3 | 33 |

[1] A / indicates no animals were present in that category

**Table S2. Parvovirus screening results for mink.**

| | **AMDV** | | | **Protoparvoviruses** | | | **Co-infection** |
|---|---|---|---|---|---|---|---|
| | Positive N (%) | Negative N (%) | p | Positive N (%) | Negative N (%) | p | |
| TOTAL (N=77) | 32 (41.6) | 45 (58.4) | | 5 (6.5) | 72 (93.5) | | |
| 2017 (N=21) | 8 (38.1) | 13 (61.9) | | 1 (4.8) | 20 (95.2) | | |
| 2018 (N=40) | 17 (42.5) | 23 (57.5) | 0.7-0.9 | 1 (2.5) | 39 (97.5) | 0.07-0.7 | |
| 2019 (N=16) | 7 (43.8) | 9 (56.2) | | 3 (18.8) | 13 (81.2) | | X |
| **South-east VI (N=60)** | 24 (40) | 36 (60) | | 4 (6.7) | 56 (93.3) | | |
| Cowichan River Valley (N=6) | 1 (16.7) | 5 (83.3)* | | 2 (33.3) | 4 (66.7) | | |
| North Cowichan (N=7) | 3 (75) | 4 (25) | | 2 (28.6) | 5 (71.4) | | X |
| Mesachie Lake (N=18) | 7 (38.9) | 11 (61.1)* | | 0 (0) | 18 (100) | | |
| Copper Canyon (N=11) | 6 (54.5) | 5 (45.5) | | 0 (0) | 11 (100) | | |
| Ladysmith-Mckay Peak (N=14) | 5 (35.7) | 9 (64.3)* | | 0 (0) | 14 (100) | | |
| Coombs/Blueberry Road (N=4) | 2 (50) | 2 (50) | | 0 (0) | 4 (100) | | |
| **South-west VI (3)** | 0 (0) | 3 (100) | | 0 (0) | 3 (100) | | |
| French Creek Headwaters (N=3) | 0 (0) | 3 (100)* | | 0 (0) | 3 (100) | | |
| **Middle-island VI (N=9)** | 8 (88.9) | 1 (11.1) | | 0 (0) | 9 (100) | | |
| Great Central Lake (N=9) | 8 (88.9) | 1 (11.1) | **<0.02** | 0 (0) | 9 (100) | | |
| **North-east VI (N=4)** | 0 (0) | 4 (100) | | 1 (33.3) | 3(66.7) | | |
| Campbell River (N=4) | 0 (0) | 4 (100)* | | 1 (33.3) | 3 (66.7) | | |
| **Quadra Island (N=1)** | 0 (0) | 1 (100) | | 0 (0) | 1 (100) | | |
| Female (N=21) | 8 (38.1) | 13 (61.9) | 0.7 | 0 (0) | 21 (100) | 0.2 | |
| Male (N=56) | 24 (42.9) | 32 (57.1) | | 5 (8.9) | 51 (91.1) | | X |
| Adult (N=66) | 30 (45.5) | 36 (54.5) | 0.1 | 5 (7.6) | 61 (92.4) | 0.5 | X |
| Juvenile (N=11) | 2 (18.2) | 9 (81.8) | | 0 (0) | 11 (100) | | |
| Excellent (N=6) | 2 (40) | 4 (60) | | 1 (20) | 5 (80) | | |
| Good (N=32) | 11 (34.4) | 21 (65.6) | 0.1-0.8 | 4 (12.5) | 28 (87.5) | 0.07-0.9 | X |
| Fair (N=29) | 16 (55.2) | 13 (44.8) | | 0 (0) | 29 (100) | | |
| Poor (N=10) | 3 (30) | 7 (70) | | 0 (0) | 10 (100) | | |
| Enlarged spleen (N=8) | 2 (25) | 6 (75) | 0.4 | | | | |
| Non-enlarged spleen (N=69) | 30 (43.5) | 39 (56.5) | | | | | |
| | Average (SD) | Average (SD) | | Average (SD) | Average (SD) | | |
| Weight (kg) | 0.73 (0.23) | 0.71 (0.26) | 0.7 | 0.99 (0.20) | 0.70 (0.24) | **0.01** | 0.89 |
| Length (mm) | 558.03 (42.66) | 537.93 (65.05) | 0.1 | 509.60 (114.49) | 548.83 (51.80) | 0.1 | 576.00 |
| Chest girth (mm) | 158.84 (23.34) | 159.27 (24.57) | 0.9 | 173.60 (16.21) | 158.08 (24.12) | 0.2 | 179.00 |

* Prevalence was significantly lower than in Great Central Lake area.

**Table S3. Parvovirus screening results for Vancouver Island Pacific martens.**

| | AMDV | | | Protoparvoviruses | | |
|---|---|---|---|---|---|---|
| | Positive N (%) | Negative N (%) | p | Positive N (%) | Negative N (%) | p |
| TOTAL (N=107) | 4 (3.7) | 103 (96.3) | | 1 (0.9) | 106 (99.1) | |
| 2016 (N=76) | 3 (3.9) | 73 (96.1) | 0.9 | 1 (1.3) | 75 (98.7) | 0.7 |
| 2017 (N=31) | 1 (3.2) | 30 (96.8) | | 0 (0) | 31 (100) | |
| **South-east VI (N=47)** | 0 (0) | 47 (100) | | 1 (2.1) | 46 (97.9) | |
| Mesachie Lake (N=18) | 0 (0) | 18 (100) | | 0 (0) | 18 (100) | |
| Copper Canyon (N=14) | 0 (0) | 14 (100) | | 1 (7.1) | 13 (92.9) | |
| Ladysmith-Mckay Peak (N=15) | 0 (0) | 15 (100) | | 0 (0) | 15 (100) | |
| **Middle-island VI (N=5)** | 1 (20) | 4 (80) | | 0 (0) | 5 (100) | |
| Upper Taylor River (N=5) | 1 (20) | 4 (80) | | 0 (0) | 5 (100) | |
| **North-west VI (N=17)** | 0 (0) | 17 (100) | | 0 (0) | 17 (100) | |
| Fair Harbour area (N=17) | 0 (0) | 17 (100) | | 0 (0) | 17 (100) | |
| **North-east VI (N=38)** | 3 (7.9) | 35 (92.1) | | 0 (0) | 38 (100) | |
| Rupert Main (N=9) | 1 (11.1) | 8 (88.9) | | 0 (0) | 9 (100) | |
| Coho Road (N=5) | 0 (0) | 5 (100) | | 0 (0) | 5 (100) | |
| West Main (N=6) | 0 (0) | 6 (100) | | 0 (0) | 6 (100) | |
| Cluxewe Road (N=2) | 1 (50) | 1 (50) | | 0 (0) | 2 (100) | |
| Alice main (N=2) | 1 (50) | 1 (50) | | 0 (0) | 2 (100) | |
| Others (N=14) | 0 (0) | 14 (100) | | 0 (0) | 14 (100) | |
| Female (N=57) | 2 (3.5) | 55 (96.5) | 0.9 | 1 (1.8) | 56 (98.2) | 0.5 |
| Male (N=50) | 2 (4) | 48 (96) | | 0 (0) | 50 (100) | |
| Adult (N=49) | 2 (4.1) | 47 (95.9) | 0.9 | 0 (0) | 49 (100) | 0.5 |
| Juvenile (N=58) | 2 (3.4) | 56 (96.6) | | 1 (1.7) | 57 (98.3) | |
| Excellent (N=3) | 0 (0) | 3 (100) | 0.8->0.9 | 0 (0) | 3 (100) | 0.7->0.9 |
| Good (N=27) | 1 (3.7) | 26 (96.3) | | 0 (0) | 27 (100) | |
| Fair (N=70) | 3 (4.3) | 67 (95.7) | | 1 (1.4) | 69 (98.6) | |
| Poor (N=7) | 0 (0) | 7 (100) | | 0 (0) | 7 (100) | |
| | Average (SD) | Average (SD) | | | Average (SD) | |
| Weight (kg) | 0.75 (0.14) | 0.80 (0.17) | 0.6 | 0.72 | 0.8 (0.17) | 0.6 |
| Length (mm) | 534.50 (109.85) | 550.10 (82.61) | 0.7 | 566 | 549.35 (83.56) | 0.8 |
| Chest girth (mm) | 149.50 (22.17) | 147.50 (14.15) | 0.8 | 129 | 147.75 (14.33) | 0.2 |

**Table S4. Protoparvovirus screening results for Haida Gwaii Pacific martens.**

| | **Protoparvoviruses** | | |
|---|---|---|---|
| | Positive N (%) | Negative N (%) | p |
| TOTAL (N=23) | 1 (4.3) | 22 (95.7) | |
| 2016 (N=3) | 0 (0) | 3 (100) | 0.3-0.7 |
| 2017 (N=6) | 1 (16.7) | 5 (83.3) | |
| 2018 (N=14) | 0 (0) | 14 (100) | |
| Haida Gwaii, Graham Island (N=9) | 1 (11.1) | 8 (88.9) | 0.4 |
| Haida Gwaii, Moresby Island (N=14) | 0 (0) | 14 (100) | |
| Female (N=3) | 0 (0) | 3 (100) | 0.9 |
| Male (N=20) | 1 (5) | 19 (95) | |
| Adult (N=16) | 0 (0) | 16 (100) | 0.3 |
| Juvenile (N=7) | 1 (14.3) | 6 (85.7) | |
| Excellent (N=1) | 0 (0) | 1 (100) | 0.3-0.9 |
| Good (N=13) | 0 (0) | 13 (100) | |
| Fair (N=3) | 0 (0) | 3 (100) | |
| Poor (N=6) | 1 (16.7) | 5 (83.3) | |
| | | Average (SD) | |
| Weight (kg) | 0.77 | 1.08 (0.29) | 0.3 |
| Length (mm) | 570 | 550.32 (129.12) | 0.9 |
| Chest girth (mm) | 193 | 184.82 (33.66) | 0.8 |

**Table S5. Protoparvovirus screening results for river otters.**

| | **Protoparvoviruses** | | |
|---|---|---|---|
| | Positive N (%) | Negative N (%) | p |
| TOTAL (N=22) | 7 (31.8) | 15 (68.) | |
| 2019 (N=22) | 7 (31.8) | 15 (68.2) | |
| **South-east VI (N=14)** | 5 (35.7) | 9 (64.3) | |
| North Cowichan (N=9) | 4 (80) | 5 (20) | |
| Duncan Hatchery (N=2) | 0 (0) | 2 (100) | |
| Cowichan River Valley (N=3) | 1 (33.3) | 2 (67.7) | |
| **South-west VI (N=8)** | 2 (25) | 6 (75) | |
| Sooke to Jordan River (N=8) | 2 (25) | 6 (75) | |
| Female (N=10) | 4 (40) | 6 (60) | 0.5 |
| Male (N=12) | 3 (25) | 9 (75) | |
| Adult (N=14) | 2 (14.3) | 12 (85.7) | **0.04** |
| Juvenile (N=8) | 5 (62.5) | 3 (37.5) | |
| Excellent (N=1) | 0 (0) | 1 (100) | 0.1-0.8 |
| Good (N=3) | 1 (33.3) | 2 (66.7) | |
| Fair (N=15) | 5 (33.3) | 10 (66.7) | |
| Poor (N=3) | 1 (33.3) | 2 (66.7) | |
| | Average (SD) | Average (SD) | |
| Weight (kg) | 7.92 (1.74) | 9.61 (2.07) | 0.08 |
| Length (mm) | 1194.29 (53.03) | 1249.20 (79.46) | 0.1 |
| Chest girth (mm) | 430.43 (47.45) | 446.87 (39.02) | 0.4 |

**Table S6. Percentage identity (1 - p-distance) within and between AMDV taxa identified in insular British Columbia.**

| | Within | Between | | | | | | | |
|---|---|---|---|---|---|---|---|---|---|
| **Taxa** | | **Clade 1** | **MAVI71** | **MAVI78** | **Clade 2** | **Clade 3** | **Clade 4** | **Clade 5** | **Clade 6** |
| **Clade 1** | 99.1 | | | | | | | | |
| **MAVI71** | / | 95.1 | | | | | | | |
| **MAVI78** | / | 94.5 | 95.3 | | | | | | |
| **Clade 2** | 99.2 | 94.7 | 96 | 96.6 | | | | | |
| **Clade 3** | 98.3 | 93.8 | 94.3 | 95.8 | 97 | | | | |
| **Clade 4** | 99.3 | 93.6 | 93.6 | 95.3 | 95.9 | 97 | | | |
| **Clade 5** | 99.8 | 93.2 | 93.6 | 94.9 | 95.4 | 95.8 | 96.1 | | |
| **Clade 6** | 97.1 | 91.5 | 91.8 | 92.3 | 93.7 | 94 | 93.7 | 94.4 | |
| **Clade 7** | 99.4 | 94.2 | 94.1 | 95.1 | 95.9 | 95.8 | 95.1 | 95.4 | 93.7 |

**Table S7. Amino acid mutations in VP2 of the prototype strains (FPV, CPV, CPV-2a, CPV-2b and CPV-2c) and of the viruses characterized in this study.**

| Residue | FPV | Study FPV | CPV | Study CPV | CPV-2a | CPV-2b | CPV-2c |
|---|---|---|---|---|---|---|---|
| **80** | K | K | R | R | R | R | R |
| **87** | M | M | M | L | L | L | L |
| **93** | K | K | N | N | N | N | N |
| **101** | I/T | T | I | T | T | T | T |
| **103** | V | V | A | A | A | A | A |
| **305** | D | D | D | H | Y | Y | Y |
| **323** | D | D | N | N | N | N | N |
| **426** | N | N | N | N | N | D | E |
| **564** | N | N | S | S | S | S | S |
| **568** | A | A | G | G | G | G | G |

**Figure S1. Phylogenetic analysis of the complete amdoparvoviral NS1 gene.** The phylogenetic tree was obtained with the neighbor-joining method [1], based on the Tamura 3-parameter model [2], identified as the best-fitting model after the model test analysis, using MEGA 7 [3]. The outcome of the bootstrap analysis [4] is shown next to the nodes. Strains identified in BC are labelled with circles colored corresponding to the host in which they were identified (black for VI mink, blue for farmed mainland BC mink, red for martens, orange for BC skunks). A subtree derived from the main tree showing the strains obtained in this study is shown on the right.

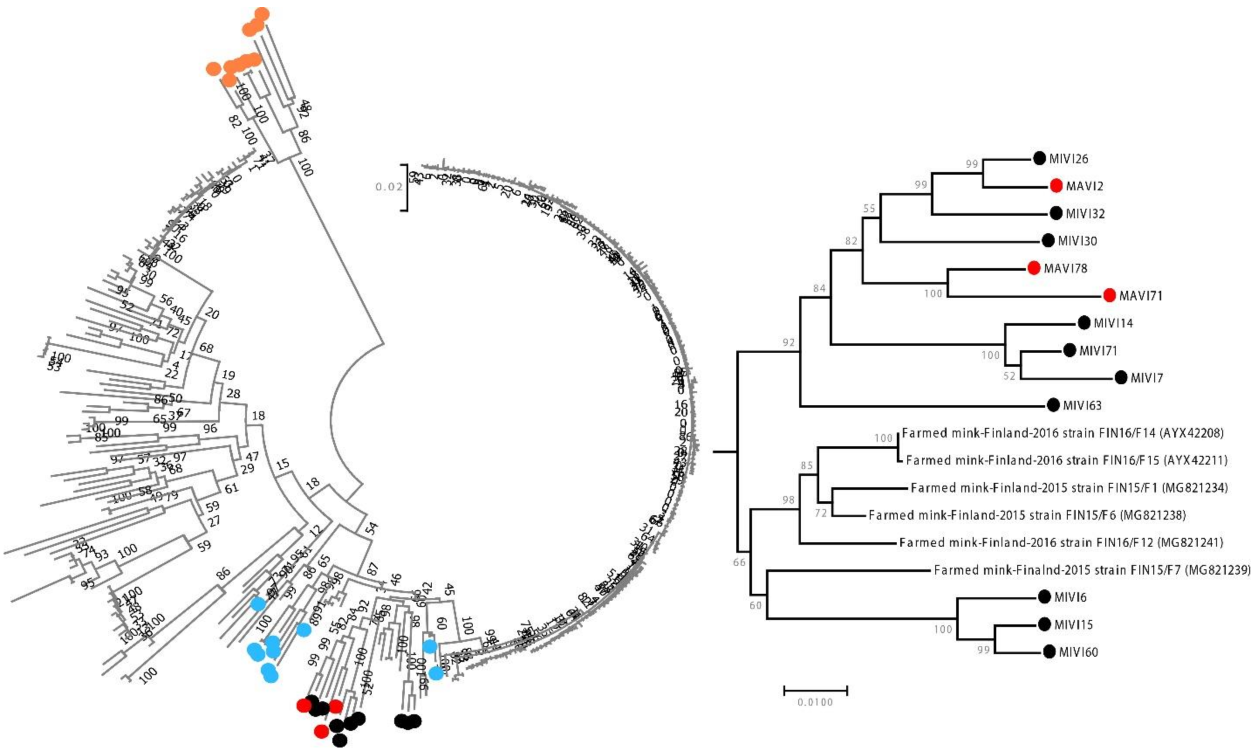

**Figure S2. Phylogenetic analysis of the complete amdoparvoviral VP2 gene.** The phylogenetic tree was obtained with the neighbor-joining method [1], based on the Tamura Nei model [5], identified as the best-fitting model after the model test analysis, using MEGA 7 [3]. The outcome of the bootstrap analysis [4] is shown next to the nodes. Strains identified in BC are labelled with circles colored corresponding to the host in which they were identified (black for VI mink, red for martens, orange for BC skunks). A subtree derived from the main tree showing the strains obtained in this study is shown on the right.

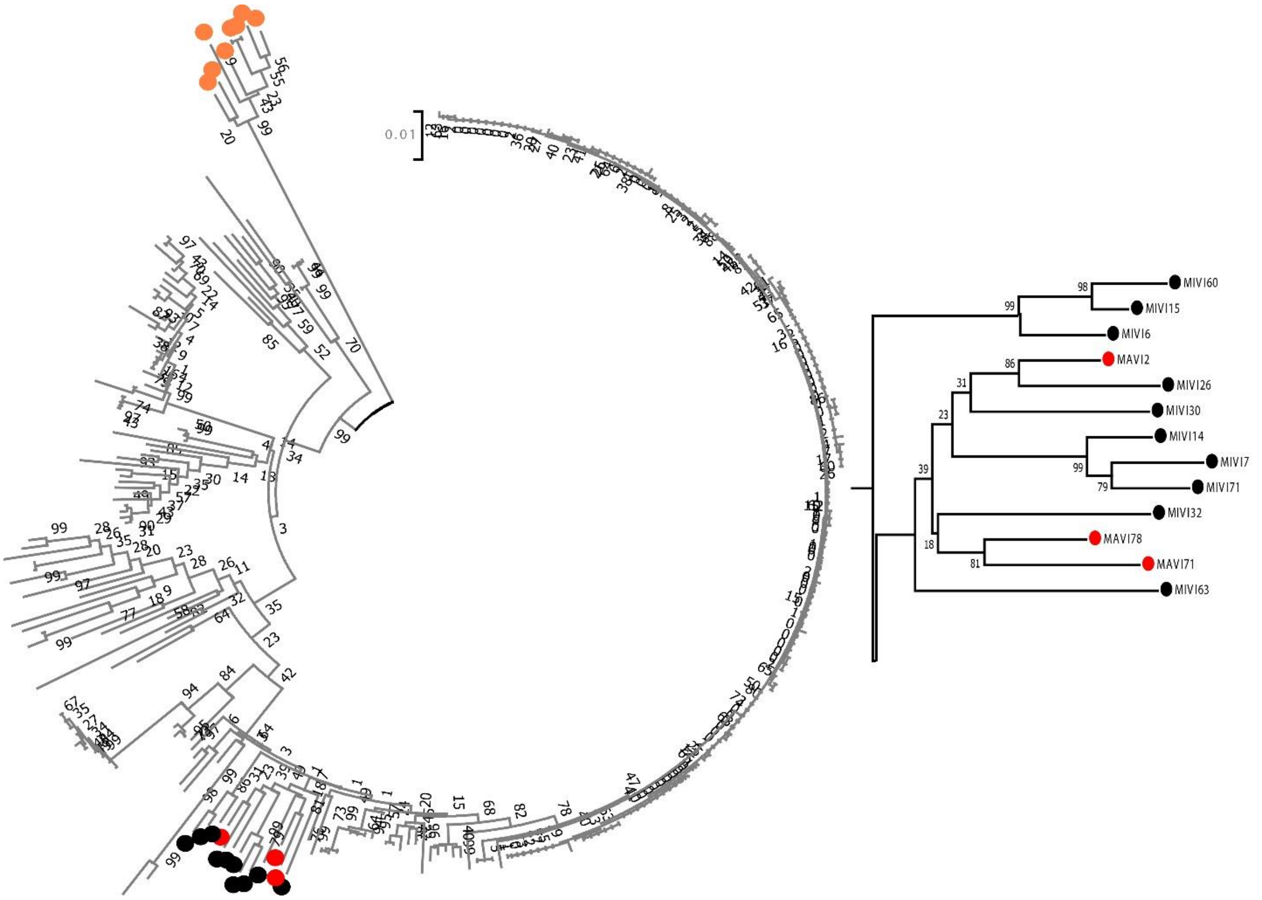

**Figure S3. Phylogenetic analysis of the complete protoparvoviral NS1 gene.** The phylogenetic tree was obtained with the neighbor-joining method [1], based on the Tajima-Nei model [6], identified as the best-fitting model after the model test analysis, using MEGA 7 [3]. The outcome of the bootstrap analysis [4] is shown next to the nodes. Strains identified in BC are labelled with circles colored corresponding to the host in which they were identified (black for otters, red for mink, purple for VI marten, orange for HG marten, blue for raccoons). Subtrees derived from the main tree showing the strains obtained in this study are shown on the left. Subtrees are indicated by letters that indicate the location of these branches on the main tree.

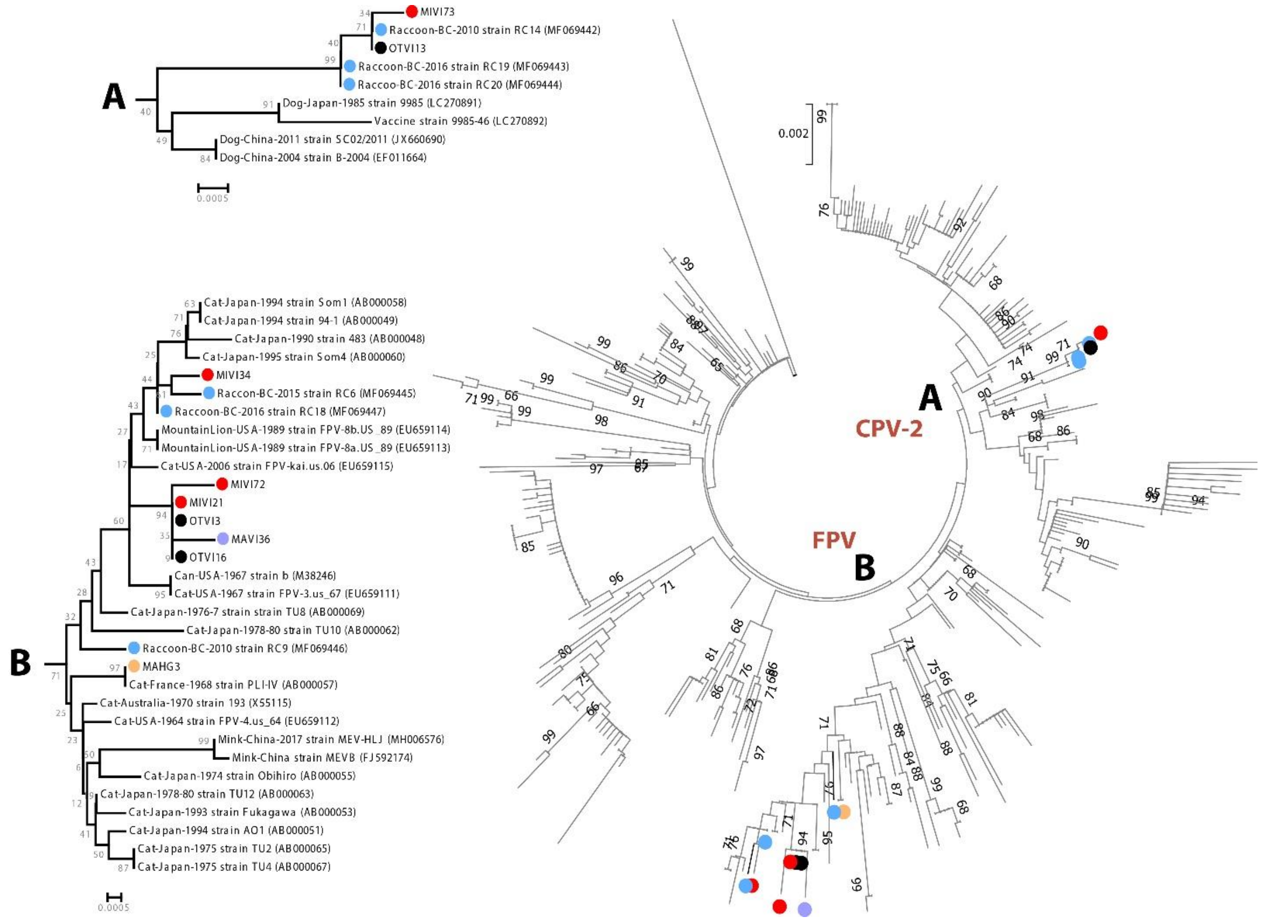

**Figure S4. Phylogenetic analysis of the complete protoparvoviral VP2 gene.** The phylogenetic tree was obtained with the neighbor-joining method [1], based on the Tamura 3-parameters model [2], identified as the best-fitting model after the model test analysis, using MEGA 7 [3]. The outcome of the bootstrap analysis [4] is shown next to the nodes. Strains identified in BC are labelled with circles colored corresponding to the host in which they were identified (black for otters, red for mink, purple for VI martens, orange for HG marten, blue for raccoons). Subtrees derived from the main tree showing the strains obtained in this study are shown on the top and on the right. Subtrees are indicated by letters that indicate the location of these branches on the main tree.

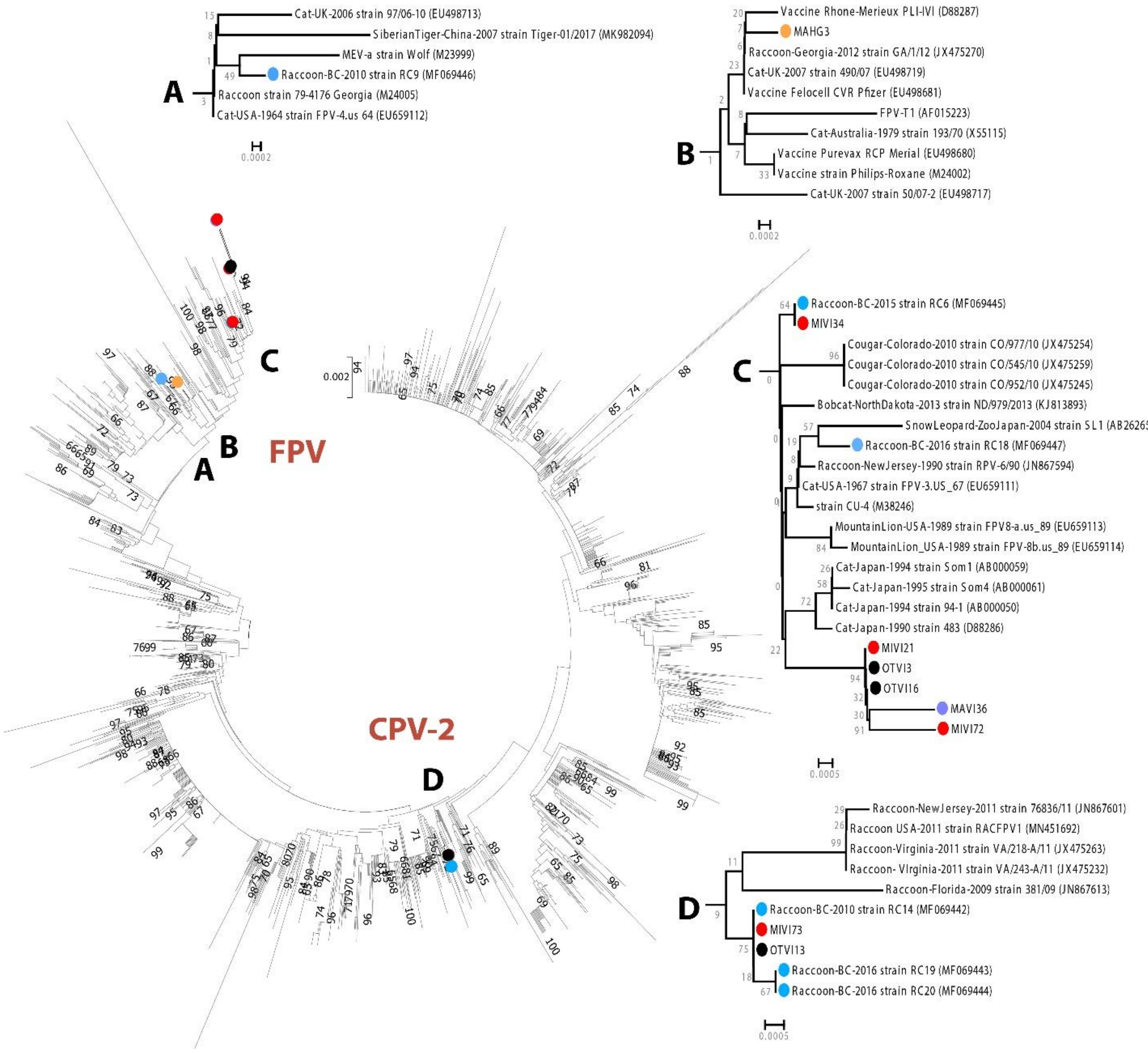

1. Saitou, N.; Nei, M. The neighbor-joining method: a new method for reconstructing phylogenetic trees. *Mol Biol Evol* **1987**, *4*, 406–425.
2. Tamura, K. Estimation of the number of nucleotide substitutions when there are strong transition-transversion and G+C-content biases. *Mol Biol Evol* **1992**, *9*, 678–687.
3. Kumar, S.; Stecher, G.; Tamura, K. MEGA7: Molecular Evolutionary Genetics Analysis version 7.0 for bigger datasets. *Mol Biol Evol* **2016**, *33*, 1870–1864.
4. Felsenstein, J. Confidence limits on phylogenies: an approach using the bootstrap. *Evolution* **1985**, *39*, 783.
5. Tamura, K.; Nei, M. Estimation of the number of nucleotide substitutions in the control region of mitochondrial DNA in humans and chimpanzees. *Mol Biol Evol* **1993**, *10*, 512–526.
6. Tajima, F.; Nei, M. Estimation of evolutionary distance between nucleotide sequences. *Mol. Biol. Evol.* **1984**, *1*, 269–285.
